# Levels and trends of demographic indices in southern rural Mozambique: evidence from demographic surveillance in Manhiça district

**DOI:** 10.1186/1471-2458-6-291

**Published:** 2006-11-30

**Authors:** Ariel Q Nhacolo, Delino A Nhalungo, Charfudin N Sacoor, John J Aponte, Ricardo Thompson, Pedro Alonso

**Affiliations:** 1 Centro de Investigação em Saúde da Manhiça, Ministério de Saúde, Manhiça, Maputo, Mozambique; 2 Instituto Nacional de Saúde, Ministério de Saúde, Maputo, Mozambique; 3 Centre de Salut Internacional, Hospital Clínic/Institut d'Investigacions Biomèdiques August Pi i Sunyer, Universitat de Barcelona, Villarroel 170, 08036 Barcelona, Spain

## Abstract

**Background:**

In Mozambique most of demographic data are obtained using census or sample survey including indirect estimations. A method of collecting longitudinal demographic data was introduced in southern Mozambique since 1996 (DSS -Demographic Surveillance System in Manhiça district, Maputo province), but the extent to which it yields demographic measures that are typical of southern rural Mozambique has not been evaluated yet.

**Methods:**

Data from the DSS were used to estimate the levels and trends of fertility, mortality and migration in Manhiça, between 1998 and 2005. The estimates from Manhiça were compared with estimates from Maputo province using the 1997 National census and 1997 Demographic and Health Survey (DHS). The DHS data were used to estimate levels and trends of adult mortality using the siblings' histories and the orphanhood methods.

**Results:**

The populations in Manhiça and in Maputo province are young (44% <15 years in Manhiça and 42% in Maputo); with reduced adult males when compared to females (all ages sex ratio of 78.7 in Manhiça and 89 in Maputo). Fertility in Manhiça is at a similar level as in Maputo province and has remained around 5 children per woman, during the eight years of surveillance in Manhiça. Although the infant mortality rate (IMR) in Mozambique has decreased during the last two decades (from 148 deaths per 1000 live births in 1980 to 101 in 2003), it has remained stable around 80 in Manhiça during the surveillance period. Adult mortality has increased both in Manhiça (probability of dying from ages 15 to 60 increased from 0.4 in 1998 to 0.6 in 2005 in Manhiça, from 0.3 in 1992 to 0.4 in 1997 in Maputo province and from 0.1 in 1980 to 0.6 in 2000 in Mozambique). Consequently, the life expectancy decreased from 53 to 46 in Manhiça and from 42 years in 1997 to 38 in 2004 in Mozambique. Migration is high in Manhiça but tends to stabilise after the movements of resettlement that followed the end of the civil war in 1992.

**Conclusion:**

The population under demographic surveillance in Manhiça district presents characteristics that are typical of southern rural Mozambique, with predominance of young people and reduction of adult males. Labour migration and excess adult male mortality are the major factors for the reduction of adult males. Mortality is high and only infant mortality has started to stabilise while adult mortality has increased, and as consequence, life expectancy has decreased. The Manhiça DSS is an adequate tool to report demographic measures for southern rural Mozambique.

## Background

In Mozambique as well as in many other sub-Saharan African countries, people are born and die before being formally registered and most of demographic data are obtained using census or sample survey including indirect estimations. However, problems with surveys and census include errors such as omissions of live births that died immediately, deaths from households that disappeared after death, multi reporting of individuals and other events when the concept of household is mixed with that of family, which is likely in most African countries, and particularly, the fact that the data are often out of date [1,2].

Since 1996, a Demographic Surveillance System (DSS) has been running in Manhiça district, Maputo province, Southern rural Mozambique. A DSS aims to produce high quality estimates, but how good is it on reporting demographic indices that are typical of Mozambique has not been evaluated yet. This paper aims to assess the extent to which this DSS yields demographic measures that are typical of southern Mozambique by comparing it's demographic indices with the 1997 National Census and the 1997 Demographic and Health Survey (DHS) data.

## Methods

The data comes from different sources namely (i) the Demographic Surveillance System (DSS) in Manhiça district, Maputo province, carried out by the Manhiça Health Research Center (CISM); (ii) the Demographic and Health Survey (DHS) carried out in Mozambique by the National Institute of Statistics and the Ministry of Health in 1997 and (iii) the II^nd ^National Census carried out in Mozambique also by the National Institute of Statistics in 1997.

The surveillance in Manhiça covered an area of 100 km^2 ^with 32856 residents in mid 1998. A resident is defined as any person who lives in the study area and expects to stay for at least the next three months. In 2002 and 2005 the DSS area was extended to include 500 km^2 ^and the population has increased to 79783 in mid 2005, representing 57% of the population in Manhiça district (table 1). During the surveillance every household is visited at least twice a year and all vital events including births, deaths and migration are recorded. Other data such as pregnancies, abortions, stillbirths and level of education are also collected during these visits [3]. The semestral visits are complemented by weekly updates by the key informants in the community and daily hospital visits, to avoid possible omissions of some events if only semestral visits are used. All the vital events are linked to the individual by the Permanent Identification Number, which is issued for every resident. This allows for an accurate follow-up avoiding duplication of individuals in the database and, most importantly, allows for estimates of accurate rates by dividing the number of events by the number of person-years of observation.

The DHS data were accessed from the Macro International website. In brief, women aged 15–49 years were interviewed on the numbers of brothers and sisters born to the same mother and their corresponding vital status. Similarly, children less than 15 years of age were asked on the survival of their parents.

The analysis comprises a description of the basic demographic measures such as sex ratio, population pyramid, mean household size, age-specific fertility rates, abortion ratio, age-and-sex-specific mortality rate including the probability of dying between ages 15 and 60 years (_45_q_15_), life expectancy at birth and migration rates from the DSS. Rate ratios, 95% Confidence Intervals (CI) and P-values of mortality and migration comparing males to females, after controlling for age in Manhiça were calculated. A Poisson regression model using the year as covariate was used to evaluate the linear trends of infant mortality, adult mortality and migration in Manhiça. The coefficient for the "linear" model estimates the change in the incidence per year increase. The significance of the linear effect was evaluated using a Likelihood Ratio Test comparing the null model with the "linear" model. In order to evaluate if the linear is sufficient to explain the differences by year, a model using the year as categorical covariate was used. These estimates were compared, where applicable, with the estimates from the DHS and Census for Maputo province and Mozambique as a whole. Estimates from DHS include _45_q_15 _using the direct siblings' survival method (based on the respondent's number of siblings ever borne to the same mother and their survival status at the time of the survey), and the orphanhood method (based on the proportions of people whose parents have died). These methods are described elsewhere [1,4].

Some of the weaknesses of this analysis include (i) differences in sizes of the areas compared (Manhiça DSS area is very small compared to Maputo province and it covers only 9.9% of the Maputo province population); (ii) differences in time-periods compared (Census = 1997, Manhiça = 1998–2005 and DHS = 1997 with time location of adult deaths rolling back to 3–15 years before the survey); (iii) the inability to measure the statistical significance of the differences or similarities observed when comparing the indices from different sources. Whereas rate ratios, 95% CI, Poisson regression for trend analysis and P-values were calculated for the Manhiça DSS data, this was not possible with the census data because the National Institute of Statistics does not deliver dataset at individual level and the indices were calculated from published data in tables. Although the DHS data was at individual level, the indirect methods used did not allow for testing the significance of the differences or similarities between its indices and that of other sources.

## Results and discussion

### 2.1. Population characteristics

Villages in Manhiça typically comprise a loose conglomeration of compounds separated by garden plots and grazing land. Houses are simple, with walls typically made of cane (84%) and conventional material (15%). About 83% of households have latrines. In towns, houses are often grouped into family compounds and surrounded by grass fences. Towns grew substantially during the civil war in the 1980s as displaced people looked for refuge [3]. Water comes mainly from community wells. With the exception of the center of the towns of Manhiça, Maragra, Palmeira and Ilha Josina, which has a 24-hours public electricity service, the rest of the area relies on more traditional systems for lighting. These characteristics are common in many other areas of southern rural Mozambique [5].

The population under surveillance has increased in part due to the expansion of the study area, but the sex ratio, mean household size and the percentage of households headed by females remained constant, indicating that the newly incorporated areas were very similar to the older ones (table 1). The population growth rate within the DSS between 1997 and 2001 was 3.8%, which is 65% higher than the rate in Maputo province in 2002 (2.3%) [6]. The national population growth rate for 1997 and 2003 was 1.7% and 2.3% respectively [7].

**Figure 1:**
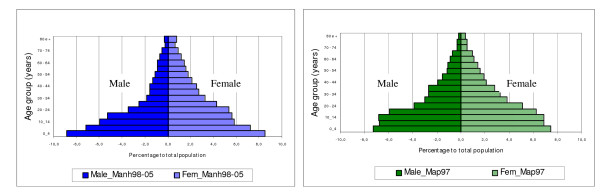
**Population pyramids, Manhiça 1998–2005 and Maputo province, 1997**. Calculated from the Manhiça DSS data and the 1997 census data [16].

The age and sex structure of the Manhiça population is typical of Maputo province and Mozambique as well as other sub-Saharan African countries, with predominance of young people and very few people in old ages (54% are <20 years old; 40%, 20–64 years old; and 5%, >65 years old (Figure 1). A particular feature of the population in southern Mozambique is the reduction of the proportion of adult males from age 20 onwards. This may be due to migration looking for employment in Maputo city and the neighbouring South Africa. The sex ratio in Manhiça during the period 1998–2005 was lower than in Maputo province (79.3% vs 89.1%), and female-headed households accounted for 32% (table 1). Sex ratio was calculated by dividing the number of males by the number of females and multiplied by 100. In Manhiça DSS, a household is defined as a group of people living under the "same roof", sharing food and other expenses and acknowledge one of them as their leader. In most of the cases the head is a man (71% in Maputo City and 80% in Sub-Saharan Africa), although it is known that underestimation of proportion of households headed by females may occur [8].

### 2.2. Fertility

**Figure 2:**
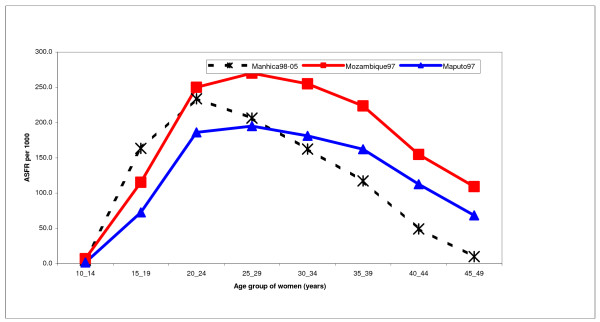
**Age-specific fertility rates, Manhiça 1998–2005; Maputo province, 1997 and Mozambique, 1997**. Calculated from the Manhiça DSS data and the 1997 census data [16]. Manhiça98-05 – age-specific fertility rate per 1000 in Manhiça DSS area, 1998–2005. Maputo97 – age-specific fertility rate per 1000 in Maputo province, 1997. Mozambique97 – age-specific fertility rate per 1000 in Mozambique, 1997.

Table 2 shows the basic fertility measures and related data and figure 2 compares age-specific fertility rates in Manhiça, Maputo province and Mozambique as a whole. Pregnancies are registered at home during the semestral updates that are complemented by daily registration at antenatal clinics. These pregnancies are followed until the end (whether abortion or stillbirth or live birth). Every woman in reproductive age that has no pregnancy or abortion or stillbirth or live birth registered during the last two years is interviewed on these reproductive events to avoid omissions. Abortion data for comparison in Mozambique is rare, but WHO estimates indicates that the incidence of unsafe abortion in Mozambique in 2000 was more than 30 unsafe abortion per 1000 women aged 15–44 years [9]. A thorough research on the meaning of the observed decrease of abortion ratio in Manhica is required.

Age-specific fertility rate is a measure that shows the variation of fertility by age of women; is obtained by dividing the number of children born to mothers in specific age by the number of women in that age. The patterns of fertility by age are similar, but women in Manhiça appears to start childbearing at very young ages and stop earlier than in Maputo province and Mozambique as a whole.

### 2.3. Mortality

**Figure 3:**
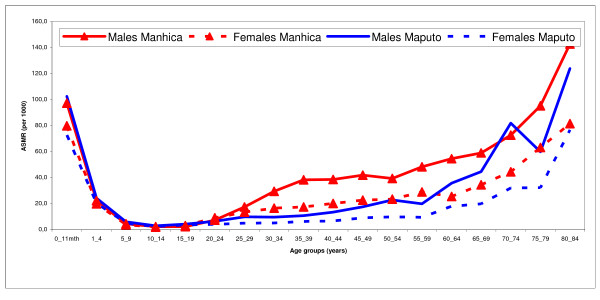
**Mortality rates by age and sex in Manhiça 1998–2005 and Maputo province, 1997**. Calculated from the Manhiça DSS data and the 1997 census data [16]. Males Manhica – age-specific mortality rate for males in Manhiça DSS area, 1998–2005. Females Manhica – age-specific mortality rate for females in Manhiça DSS area, 1998–2005. Males Maputo – age-specific mortality rate for males in Maputo province, 1997. Females Maputo – age-specific mortality rate for females in Maputo province, 1997. Note: Mortality rate ratio comparing males to females after controlling for age in Manhiça was 1.427, 95% CI = 1.291 - 1.578 and P < 0.001.

Table 3 shows the basic mortality measures and related data for Manhiça DSS, Maputo province and Mozambique as a whole. The infant mortality rate (IMR) has remained stable between 1998 and 2005, although there has been a year-to-year variation with peaks when IMR was 90 deaths per 1000 live births. A similar pattern was observed on under-five mortality but the adult mortality has been constantly increasing and, as consequence, the life expectancy at birth has decreased. IMR represents the mortality in children less than 1 year of age; life expectancy at birth is the average number of years a newborn would live given the prevailing age-specific mortality rates [10]. Figure 3 presents age and sex-specific mortality rates in Manhiça 1998–2005 and in Maputo province in 1997. The age and sex pattern of mortality within the two areas is similar (affecting more males than females, more infants and adults than adolescents). But mortality seems to be higher in adults in Manhiça than in Maputo, particularly among males, and the gap between males and females' mortality is wider in Manhiça than in Maputo province. The mortality rate ratio for male compared to female, after controlling for age in Manhiça, during the period, was 1.427, P < 0.001 (95% CI = 1.291 - 1.578).

**Figure 4:**
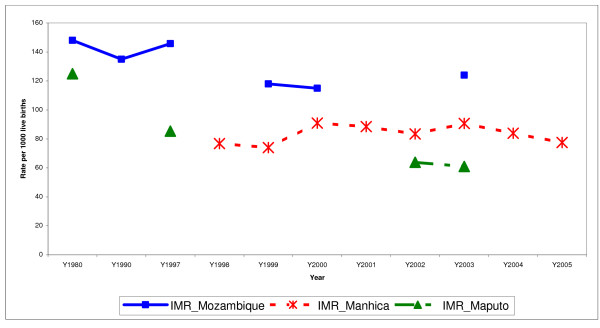
**Levels and trends of infant mortality rate, Manhiça 1998–2005, Maputo province 1980–2003 and Mozambique 1980–2003**. Manhiça DSS data and [7,16–18]. IMR_Mozambique – infant mortality rate per 1000 live births, Mozambique. Data for 2003 refers to a period 10 years before the Demographic and Health Survey date, 2003. IMR_Manhica – infant mortality rate per 1000 live births, Manhiça DSS. Note: There is no evidence that the incidence of IMR changes with the year (P-value of the Likelihood Ratio Test using the Poisson regression for linear vs null model was 0.787, and for categorical vs linear was 0.116); IMR_Maputo – infant mortality rate per 1000 live births, Maputo province. Data for 2003 refers to a period 10 years before the Demographic and Health Survey date, 2003.

**Figure 5:**
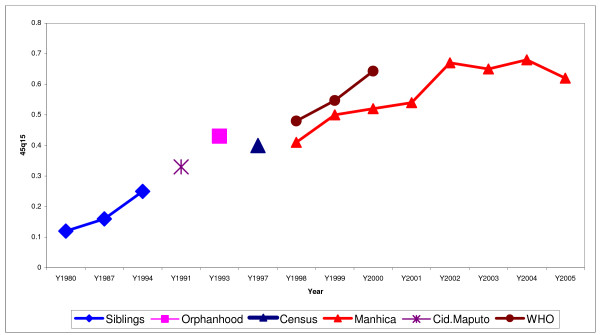
**Trends of the probability of dying between ages 15 and 60 years, Manhiça 1998–2005 and Mozambique, 1980–2000**. Siblings – _45_q_15 _estimated from the 1997 DHS data, Mozambique, using the siblings histories methods; Orphanhood – _45_q_15 _estimated from the 1997 DHS data, Mozambique, using the orphanhood method; Census – _45_q_15 _estimated from the 1997 Census data, Mozambique; Manhiça – _45_q_15 _estimated from the Manhiça DSS data. The reported trend is statistically significant (P-value of the Likelihood Ratio Test using the Poisson regression was <0.001 for both linear vs null models and categorical vs linear models); Cid.Maputo – _45_q_15 _estimates for Maputo city, 1994 [19]; WHO – _45_q_15 _estimates for Mozambique from WHO reports [11–13].

Whereas according to the DSS, IMR in Manhiça has remained stable over the last decade (P-value for the Likelihood Ratio Test using the Poisson regression for linear vs null models was 0.787, and for categorical vs linear models was 0.116), it appears to have decreased in Maputo province and Mozambique as a whole. IMR in Maputo province has decreased from 91.7 in 1997 to 63.9 in 2002 [6]; and at nation-wide level has decreased from 148 in 1980 to 101 in 2003 (table 3 and figure 4). But while infant mortality has decreased, the adult mortality (_45_q_15_) has increased from 0.4 in 1998 to 0.6 in 2005 in Manhiça (P-value for the Likelihood Ratio Test using the Poisson regression was <0.001 for both linear vs null model and categorical vs linear), and from 0.1 in 1980 to 0.6 in 2000 in Mozambique (figure 5). This increase in adult mortality is also observed in the neighbouring countries such as Swaziland, where the _45_q_15 _increased from 0.25 in 1998 to 0.61 in 2000; Lesotho, from 0.33 to 0.65; South Africa, from 0.45 to 0.53; and Namibia, from 0.44 to 0.68 [11-13]. This worsening adult mortality in Southern Africa may be explained by the AIDS epidemic [14].

### 2.4 Migration

Migration is a very important component of population change in Mozambique, but little is known about its levels and trends. Migration is the determinant of population change more difficult to study. The difficulties start with defining who is a migrant and, since migration involves two distinct places (origin and destination), one need to take into account the distance or boundaries between these places, the duration of the stay and the intention. The census and sample surveys, which are the common sources of data, track only part of these movements [10].

In Manhiça an out-migrant is a resident who leaves the DSS area for three or more months. Immigration occurs when a person enters the area to live for at least three months (include return migration). Should he move from a household to live in another household for three months or more within the DSS area he or she is regarded as internal migrant. The definition of migration in Manhiça is based on a combination of the intention of the move and the duration of the stay to avoid the ambiguity on temporary absence and temporary visits, which complicates the definition of migration events. Thus, anyone who comes into the DSS to visit his relatives, rather than to live but who stays for three months or more he is counted as an immigrant. Conversely, any person who reportedly comes to live in the area is registered as an immigrant resident immediately, and keeps this status in the database even if he subsequently leaves within three months. Irrespective of his/her economic contribution, anyone who never stays within the area for more than three months is not counted as resident. All the vital events and migration are linked to the individual by the Permanent Identification Number, which allowed estimates of rates by dividing the number of events by the number of person-years of observation. The migration rates for Maputo province were calculate by National Institute of Statistics [15] using the census data, comparing the reported place of residence five years before the census (1992), one year before the census (1996) and the place of residence at census time (1997).

**Figure 6:**
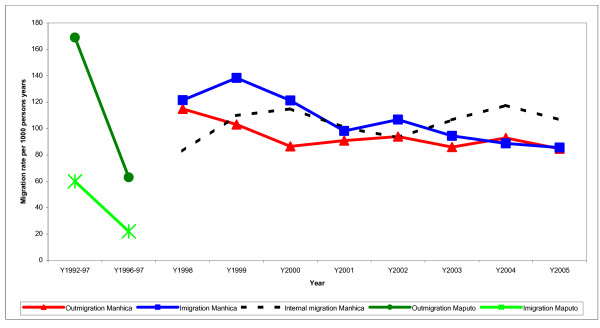
**Levels and trends of migration, Manhiça DSS, 1998 to 2005 and Maputo province, 1992–1997**. Outmigration Manhiça – emigration rate per 1000 person-years, both sexes, Manhiça DSS. Imigration Manhiça – immigration rate per 1000 person-years, both sexes, Manhiça DSS. Internal migration Manhica – internal migration rate per 1000 person-years, both sexes, Manhiça DSS. The reported trend is statistically significant (P-value for the Likelihood Ratio Test using the Poisson regression for both linear vs null model and categorical vs linear models was <0.001). Outmigration Maputo – emigration rate per 1000, both sexes, Maputo province. Imigration Maputo – immigration rate per 1000, both sexes, Maputo province. The rate for 1992–97 in Maputo province were calculated by the Instituto Nacional de Estatistica [15], by comparing place of residence in 1992 and place of residence in 1997. The rates for 1996–97 were calculated by comparing place of residence in 1996 and place of residence in 1997.

The level of migration in Manhiça (1998–2005) appears to be higher than in Maputo province in 1996–97 but both in Manhiça and in Maputo, migration has decreased during the last decade (figure 6). The P-value for the Likelihood Ratio Test using the Poisson regression indicates that the rates for emigration, immigration and internal migration in Manhiça were significantly different by year (p < 0.001). The population may be becoming less mobile than before as peace has consolidated and the resettlement movements are being completed.

**Figure 7:**
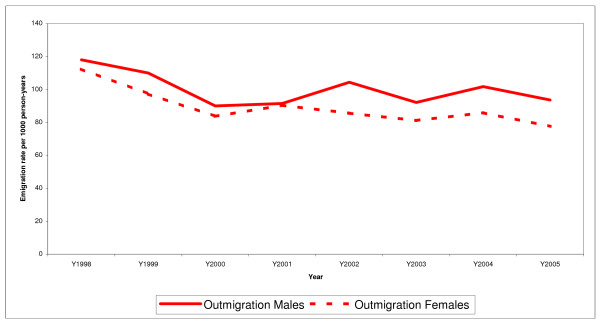
**Levels and trends of emigration by sex, Manhiça, 1998 to 2005**. Calculated from the Manhiça DSS data. Outmigration Males – emigration rate per 1000 person-years, males, Manhiça DSS area. Outmigration Females – emigration rate per 1000 person-years, females, Manhiça DSS area. Emigration rate ratio comparing males to females, after controlling for age was 1.173, 95% CI = 1.119 - 1.229 and P < 0.001.

**Figure 8:**
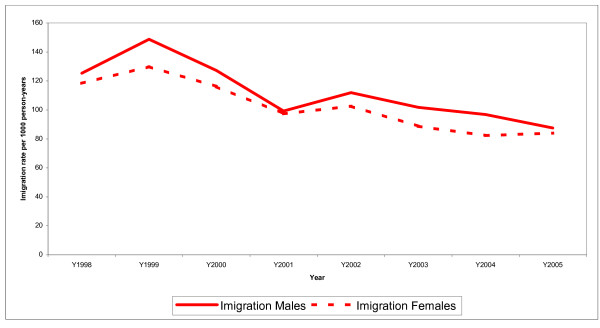
**Levels and trends of immigration by sex, Manhiça, 1998 to 2005**. Calculated from the Manhiça DSS data. Imigration Males – immigration rate per 1000 person-years, males Manhiça DSS. Imigration Females – immigration rate per 1000 person-years, females Manhiça DSS. Immigration rate ratio comparing males to females, after controlling for age was 1.132, 95% CI = 1.080 - 1.119 and P = 0.007.

**Figure 9:**
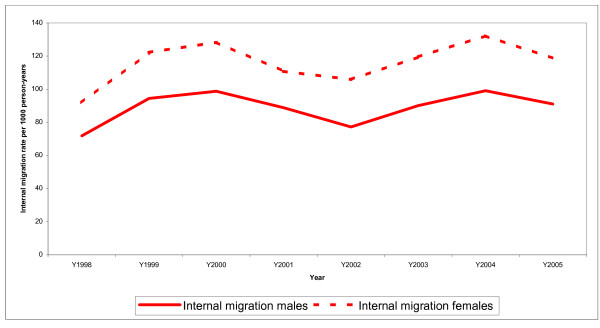
**Levels and trends of internal migration by sex, Manhiça, 1998 to 2005**. Calculated from the Manhiça DSS data. Internal migration males – internal migration rate per 1000 person-years, males Manhiça DSS. Internal migration females – emigration rate per 1000 person-years, females Manhiça DSS. Internal migration rate ratio comparing males to females, after controlling for age was 0.734, 95% CI = 0.701 - 0.768 and P < 0.001.

**Figure 10:**
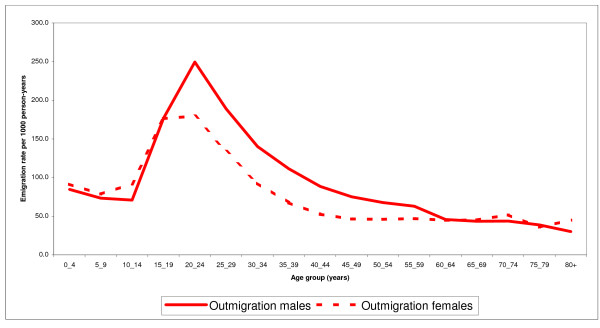
**Age-and-sex-specific emigration rates, Manhiça DSS 1998–2005**. Calculated from the Manhiça DSS data. Outmigration males – age-specific emigration rate per 1000 person-years, males. Outmigration females – age-specific emigration rate per 1000 persons-years, females.

**Figure 11:**
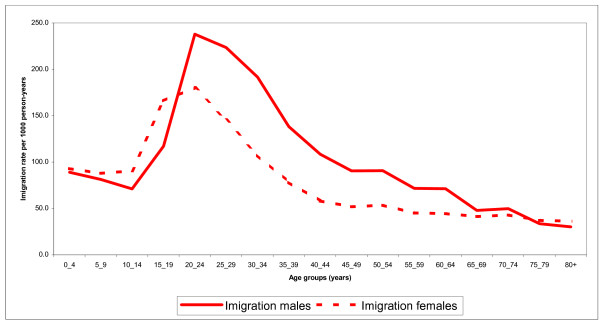
**Age-and-sex-specific immigration rates, Manhiça DSS 1998–2005**. Calculated from the Manhiça DSS data. Imigration males – age-specific immigration rate per 1000 person-years, males. Imigration females – age-specific immigration rate per 1000 persons-years, females.

**Figure 12:**
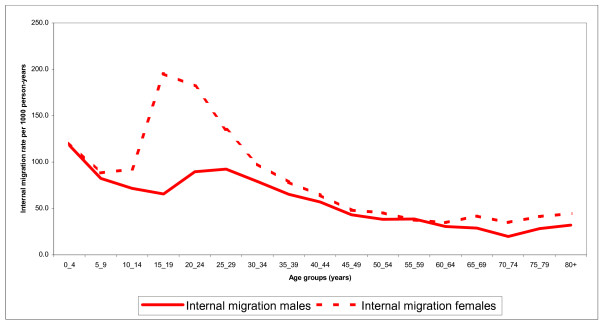
**Age-and-sex-specific internal migration rates, Manhiça DSS 1998–2005**. Calculated from the Manhiça DSS data. Internal migration males – age-specific internal migration rate per 1000 person-years, males. Internal migration females – age-specific internal migration rate per 1000 persons-years, females.

By sex, males appears to move more than females both out and into the DSS area, but internally women move more than men and the gap is much wider than for out and in-migration (figures 7, 8 and 9). Although figure 7 shows little differences of emigration by sex to justify the reduced adult males presented in figure 1, it is important to highlight that this little excess male emigration is added to a population where all the people who do not live (at least the immediately preceding three months) in the DSS were excluded since the beginning of the baseline DSS census (most of those excluded are adult men). The emigration rate ratio for males compared to females in Manhiça, during the period 1998 to 2005, was 1.173, P < 0.001 (95% CI = 1.119 - 1.229; immigration rate ratio was 1.132, P = 0.007 (95% CI = 1.080 - 1.184); while the corresponding figures for internal migration was 0.734, P < 0.001 (95% CI = 0.701 - 0.768). The excess female internal migration may be explained by the fact that women move often as result of family formation or dissolution. In case of a marriage or divorce is the woman who moves and such moves are usually to families living closeby, within the DSS. Second, when a girl gets pregnant her parents will force her to live with her boyfriend to take care of the pregnancy and she returns home after delivery and very often she is accompanied by her younger sister(s). This may also be the reason for why children <5 years old seems to move more internally than externally (figures 10, 11 and 12). These figures show that the adolescents and adults migrate more often than children and elderly probably because of education and employment. Reasons for migration are not recorded under the surveillance, and this interpretation is based on the general knowledge of the behaviour of the DSS population.

The preferred destinations for emigrants from Manhiça are the Maputo city (35.6%), followed by South Africa (19.6%), other districts within Maputo province (19%) and other provinces. Immigrants are mostly from Maputo City (40.3%), Maputo province (19.8%), South Africa (15.6%) and other provinces.

## Conclusion

The population under demographic surveillance in Manhiça district presents characteristics that are typical of southern rural Mozambique, with predominance of young people and reduction of adult males when compared to females. This reduction of adult males may represent the impact of the historical pattern of migration in southern rural Mozambique, where a work-related flow of males to Maputo city and South Africa has been kept for centuries. Despite efforts carried out to improve the health services in Mozambique, mortality is still high and only infant mortality has remained stable while adult mortality has increased, and the life expectancy has worsened over the last two decades. The pattern of mortality by age and sex in Manhiça is typical of developing countries that are heavily affected by the AIDS epidemic. The Manhiça DSS is an adequate tool to report demographic measures for southern rural Mozambique.

## Competing interests

The author(s) declare that they have no competing interests.

## Authors' contributions

AN is in charge of the DSS in Manhiça and has designed and carried out the analytical plan, including data cleaning and interpretation and manuscript writing. DN and CS have assisted AN in fieldwork supervision, data cleaning and drafting of the manuscript. JA, RT and PA designed the Manhiça DSS, including the data collection and supervision systems, and have contributed to the concept of this analysis and reviewed critically the manuscript for intellectual content. All authors have read and approved the final manuscript.

## Pre-publication history

The pre-publication history for this paper can be accessed here:



## Figures and Tables

**Table 1:** Population characteristics in Manhiça DSS (1998–2005), Maputo province (1997–2005) and Mozambique (1997–2005)

**Population characteristics**	**1998**	**1999**	**2000**	**2001**	**2002**	**2003**	**2004**	**2005**
DSS, mid yr pop.	32856	34674	37012	38163	56262	71950	73847	79783
DSS, sex ratio*	79.3	80.1	81.2	82.7	80.3	77.6	78.6	77.9
DSS, household size**	3.3	3.4	3.6	3.7	3.9	4	4.2	4.3
DSS, % female headed	32.4	32.0	31.9	31.8	28.7	29.4	29.3	29.9
Maputo prov., sex ratio*	89.9	90.7	91.4	92.0	92.5	93.0	93.4	93.8
Mozambique, sex ratio*	92.2	92.3	92.4	92.6	92.7	92.9	93.0	93.2

Sources: Manhiça DSS data and [20].

* Calculated by dividing the number of males by the number of females and multiplied by 100.

** Household size – the average number of people per household.

**Table 2:** Fertility in Manhiça DSS, 1998–2005; Maputo province and Mozambique

**Fertility**	**1998**	**1999**	**2000**	**2001**	**2002**	**2003**	**2004**	**2005**
DSS, Abortion ratio*	67.1	61.9	37.5	47.5	39.9	34.1	27.4	23.9
DSS, Still birth ratio*	31.3	22.0	30.3	40.2	22.6	22.4	27.1	21.3
DSS, Total Fertility Rate (TFR)	5.2	4.9	4.9	4.3	4.6	4.8	4.6	4.8
Maputo, TFR**	5^a^					4.1^c^		
Mozambique, TFR	6^a^		5.9^b^			5.5^c^		

Sources: Manhiça DSS data.

^a^Data refer to 1997; calculated from the 1997 census [16].

^b^Source: [21].

^c^Source: [7].

*Number of abortions per 1000 live births. In Manhiça DSS, abortion is defined as the interruption of a product of conception within 7 months of gestation, irrespective of whether the interruption was spontaneous or induced or miscarriage. If the pregnancy ends after 7 months of gestation and the foetus shows no signs of life is considered stillbirth. Then we can calculate stillbirth ratio by dividing the number of stillbirths by the number of live births, multiplied by 1000.

**Total Fertility Rate (TFR) is the average number of children a woman would bear if she survived through the entire reproductive age and experienced at each age the prevailing age-specific fertility rates [10].

**Table 3:** Mortality in Manhiça 1998–2005, Maputo province 1997–2002 and Mozambique, 1997–2004

	**Manhiça DSS**								**Maputo**	**Mozambique**				
			
				_45_q_15_^b^	_45_q_15_	Life expectancy						_45_q_15_^f^	_45_q_15_^f^	
Year	Infant* M. Rate	Under five Mort. 5q0	Maternal M. ratio^a^	Male	Female	Male	Fem	All sex	Infant* M.Rate	Infant M.R*	Matern M. ratio	Male	Female	Life Expect
1997									91.7	145.7		0.51	0.46	42.3
1998	76.8	132.0	4.5	0.52	0.33	48.5	57.1	53.2						
1999	74.0	114.9	3.6	0.62	0.41	45.3	55.3	50.6		118		0.58	0.51	46
2000	90.9	169.5	3.3	0.61	0.44	43.0	50.2	47.1		115^c^		0.67	0.61	41.7
2001	88.5	184.1	8.8	0.61	0.47	41.2	48.8	45.2						
2002	83.5	136.2	12.0	0.75	0.59	38.7	46.6	42.8	63.9	128^d^				
2003	90.7	179.4	8.1	0.76	0.55	38.7	46	42.9		101^e^	7.8^d^			
2004	83.9	133.5	6.3	0.77	0.61	37.8	46.8	42.6						38.1^g^
2005	77.5	138.6	7.5	0.75	0.52	40.2	50.1	45.5						

Sources: Manhiça DSS and [16]. See other sources below.

*Infant Mortality rate per 1000 live births.

^a^Maternal mortality ratio per 1000 live births.

^b^Probability of dying between ages 15 and 60 years (_45_q_15_).

^c^Source: [18].

^d^Source: [21].

^e^Source: [7].

^f^Source: [11–13].

^g^Source: [17].

## References

[B1] Timaeus I, Garaham W (1988). Measuring adult mortality in developing countries: a review and assessment of methods. CPS Working paper N0 88-4.

[B2] Blacker J (1977). The estimation of adult mortality in Africa from data on orphanhood. Population Studies.

[B3] Alonso P, Saute F, Aponte J, Gomez-Olive F, Nhacolo A, Thompson R (2002). Manhiça Demographic Surveillance System, Mozambique. Population and health in developing countries.

[B4] Timaeus I, Zaba B, Blacker J, B Z and J B (2001). Estimation of adult mortality from data on adult siblings.. Brass Tacks Essays in Medical Demography A tribute to the memory of Professor William Brass.

[B5] (INE) INE (2003). Características sócio-económicas das comunidades rurais em Moçambique, 2002/3. Relatório final..

[B6] (INE) INE (2003). Anuário estatístico 2002, província de Maputo.

[B7] (INE) INE, (MISAU) MS (2005). Moçambique, inquérito demográfico e de saúde 2003.

[B8] (INE) INE (1999). Mulheres chefes de agregados familiares em Maputo Cidade. Cifras e realidades..

[B9] WHO (2004). Unsafe abortion: Global and regional estimates of the incidence of unsafe abortion and associated mortality in 2000..

[B10] Newell C (1998). Methods and models in demography.

[B11] (WHO) WHO (1999). The World Health Organization Report 1999. Conquering suffering, enriching humanity.

[B12] (WHO) WHO (2000). The World Health Organization Report 1999. Annex.

[B13] (WHO) WHO (2001). The World Health Organization Report 2000. Mental Health: new understandings, new hope.

[B14] Gom P, Clark S (2003). Adult mortality in the era of HIV/AIDS: Sub-Saharan Africa. In workshop on hiv/aids and adult mortality in developing countries.. UN Population Division New York, 8-13 September 2003.

[B15] (INE) INE (2000). Atlas sócio-demográfico de Moçambique.

[B16] (INE) INE (1999). II Recenseamento geral da população e habitação. Resultados definitivos..

[B17] Mozambique UNICEF (2006). Mozambique.

[B18] Census USB (2000). Data Mozambique 1997: Results from the Demographic and Health Survey..

[B19] Dgedge M, Novoa A, Macassa G, Sacarlal J, Black J, Michaud (2001). The burden of disease in Maputo City, Mozambique: registered and autopsied deaths in 1994.. Bulletin of the World Health Organization, 2001.

[B20] (INE) INE (1999). Projecções anuais da população por província e área de residência, 1997-2010. Moçambique. Serie: Estudos No 2.

[B21] Globalis (2006). Globalis-Mozambique (quoting UN common database).. http://globalis.gvu.unu.edu/indicator_detail.cfm?IndicatorID=138&Country=MZ.

